# Bioinformatic analysis of proteomics data

**DOI:** 10.1186/1752-0509-8-S2-S3

**Published:** 2014-03-13

**Authors:** Andreas Schmidt, Ignasi Forne, Axel Imhof

**Affiliations:** 1 Munich Center of Integrated Protein Science and Adolf-Butenandt Institute, Ludwig Maximilians University of Munich, 80336 Munich, Germany

## Abstract

Most biochemical reactions in a cell are regulated by highly specialized proteins, which are the prime mediators of the cellular phenotype. Therefore the identification, quantitation and characterization of all proteins in a cell are of utmost importance to understand the molecular processes that mediate cellular physiology. With the advent of robust and reliable mass spectrometers that are able to analyze complex protein mixtures within a reasonable timeframe, the systematic analysis of all proteins in a cell becomes feasible. Besides the ongoing improvements of analytical hardware, standardized methods to analyze and study all proteins have to be developed that allow the generation of testable new hypothesis based on the enormous pre-existing amount of biological information. Here we discuss current strategies on how to gather, filter and analyze proteomic data sates using available software packages.

## Background

**Figure 1:**
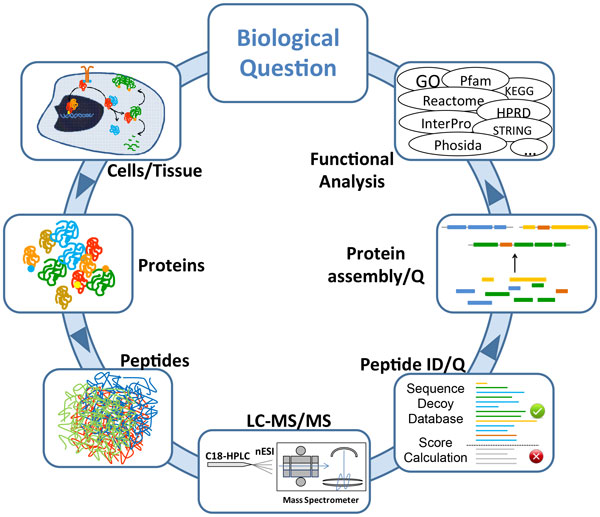
**Integrated Proteomic Workflow: Samples of interest are subjected to protein extraction and digestion**. The resulting peptides are separated by C18 chromatography and directly electrosprayed into the mass spectrometer, where their mass-to-charge ratio and fragmentation spectra is recorded. MS data is analysed to identify and quantify the detected peptides, and assemble it to proteins. Once the proteomics analysis *per se *is finished, the functional analysis of the relevant differential proteins may unmask pathways, interactions, PTM's relevant for the biological question of interest. This *in silico *information can be used to formulate new hypothesis that could be eventually used to interrogate the biological system again.

Proteins are involved in almost all physiological aspects of cellular life from the catalysis of biochemical reactions within the intermediary metabolismn to the processing and integration of internal and external signals. The misregulation of protein expression results in pathological states such as cancer, neurodegenerative diseases and metabolic imbalances. Proteins are synthesized by translating the information encoded in a RNA molecule to a polypeptide chain, which adopts a specific three dimensional structure. Proteins are subjected to a constant turnover making protein homeostasis a very important feature of their regulation. Many proteins function within large multimeric complexes that are highly dosage dependent. The recent developments in gathering large scale genomic, transcriptomic and proteomic data pose substantial challenges to the bioinformatic processing of these data, which have yet not been completely solved. In case of the proteomic investigation, the challenges occur at all levels ranging from sample preparation and data gathering over the raw data integration and database searching to the functional interpretation of large datasets. Based on a iterative strategy of proteomic analysis, data interpretation and sytstematic challenges, hypothesis can be developed and modified, which will eventually lead to the generation of new knowledge (Figure 1).

### Mass spectrometry data analysis

All proteins from a sample of interest are usually extracted and digested with one or several proteases (typically trypsin alone or in combination with Lys-C [1]) to generate a defined set of peptides. Several enrichment and fractionation steps can be introduced at protein or peptide level in this general workflow when sample complexity has to be reduced or when a specific subset of proteins/peptides should be analysed (i.e. organelle specific proteome [2,3] or substoichiometric post-translational modified peptides [4]).

The peptides obtained are subsequently analysed by liquid chromatography coupled to mass spectrometry (LC-MS). The two most common approaches here are either designed to achieve a deep coverage of the proteome (shotgun MS [5]) or to collect as much quantitative information as possible for a defined set of proteins/peptides (targeted MS [6]). During the analysis peptides eluting from the chromatography are selected according to defined rules (see below) and further fragmented within the mass spectrometer. The resulting tandem mass spectra (MS^2^) provide information about the sequence of the peptide, which is key to their identification. For a shotgun approach, no prior knowledge of the peptides present in the sample is required to define peptide selection criteria during the LC-MS analysis. Therefore, the peptides eluting from the chromatographic column are identified in a data-dependent mode [7], where continuously the N most abundant peptides at a given retention time are selected for fragmentation and their masses excluded for further selection during a defined time. By using this dynamic exclusion [8], less abundant peptides are also selected for fragmentation.

The data can be displayed as a 3-D map with the mass-to-charge ratios (m/z), retention times (RT) and intensities for the observed peptides as axis, together with fragmentation spectra (MS^2^) for those peptides that were selected during any of the data dependent cycles. The intensity of a certain peptide m/z can be plotted along the RT to obtain the corresponding chromatographic peak. The area under this curve (AUC) can be employed to quantify the corresponding peptide. On the other hand, the peptide identification is achieved through its fragmentation spectrum.

The large number of MS^2 ^spectra generated by the last generations of mass spectrometers requires automated search engines capable of identifying and quantifying the analysed peptides. It is not the aim of this review to detail the existing algorithms (see [9] for this purpose), but to give a general idea how they work and which kind of data should be expected from them. Briefly, search algorithms aim to explain a recorded MS^2 ^spectrum by a peptide sequence from a pre-defined database, returning a list of peptide sequences that fit to the experimental data with a certain probability score or false discovery rate (FDR). The databases are normally protein databases translated from genomic data [10], although other strategies like spectral libraries [11] or mRNA databases [12] have been successfully applied. A final step is then required to assemble the identified peptides into proteins, which can be challenging, in particular when dealing with redundant peptides or alternatively spliced proteins [13]. In any of these cases, several strategies have been described to reduce the false discovery rate of such matching approaches both at peptide identification and protein assembling level [14].

This general shotgun/discovery approach leads to the identification of thousands of proteins with a dynamic range of 10^4^-10^5 ^[15] within a complete cellular lysate. However, this method presents still two main drawbacks: sensitivity and reproducibility. Normally, complete coverage of proteins and complexes involved in the same signaling pathway or belonging to the same functional family is not achieved. Additionally, reproducibility in protein identification among replicates can vary between 30 and 60% [16,17]. These limitations have been successfully addressed by the so-called targeted proteomics [6]. This approach is based on a general method called selected reaction monitoring (SRM), where predefined peptides at scheduled RT are selected and fragmented, and two or three fragments monitored. Due to the increased scan speed and mass window selectivity of the current mass analyzers, SRM can be simultaneously performed on multiple analytes. This capability lead to the multiplexing of SRMs in a method called multiple reaction monitoring (MRM). The multiplexing capability have been used to quantify several hundreds of proteins in a broad dynamic range, down to proteins present at very low copy number in the cell (~50 copies/cell) in the background of the whole range of protein concentration in eukaryotic cells [18,19].

The AUC of the monitored fragments can then be used for quantification. By spiking the peptide mixture with isotopically labelled standard peptides, such targeted approaches can also be used to determine absolute rather than relative quantitation levels of proteins [20] or posttranslational modifications [21]. However, as previous knowledge about the proteins is required, such targeted approaches are usually performed in combination or subsequent to a shotgun approach. Similarly to the genomic data, shot gun proteomic studies can also be uploaded to dedicated proteome repositories [22], which can also be used for database searching. The cooperation of the largest repositories PRIDE, Proteome Commons and Peptide Atlas within the Proteome Exchange project http://www.proteomeexchange.org allow direct access to most of the stored proteomic datasets and provides a highly valuable source for bioinformatics data mining [23-25].

### GO Term identification and enrichment analysis

The output of a proteome analysis either in a shotgun approach or a more targeted method is usually a long list of identified factors, that have a probability score and ideally also a quantitative value associated with them. In order to understand and interprete these data and to generate testable hypothesis on the systemic response of the proteome to a challenge, the list has to be further classifiied and filtered. The first step for a functional analysis of a large protein list is to connect the protein name to a unique identifier. While gene names have been standardized, protein names can differ between different databases and even releases of the same database. Although many of the large databases have been curated throughout the recent years, this can pose quite a bioinformatic challenge and can lead to a substantial loss of information. Several web-based algorithms exist to connect protein names to their corresponding gene names, such as PICR or CRONOS [26];[27]. However some functional databases like the Uniprot knowledge base, Ensembl or the outdated IPI number (International Protein Index)[28-30] can use protein identifiers as input.

A first step for functional interpretation of the resultant protein list is to connect the protein identifier with its associated Gene Ontology terms (http://www.geneontology.org, [31]). Introduction of the Gene Ontology helped to overcome the redundancy in terminology for biological processes [32]. Thereby, genes are associated to hierarchically clustered, functional terms that describe the "biological process", "molecular function" or "cellular component" which have a unique identification number. A specific GO term can be related to more than one parent terms, as long as the whole structure resembles an acyclic graph. This list of terms is not yet complete and changes with new discoveries, making GO terms redundant or obsolete. Another drawback of the use of GO terms for functional annotations is the fact that most (95%) of the GO terms annotations are done computational, while the minority is manually curated and based on experimental details [32]. For single proteins the simplest way to perform a GO term annotation is to look up the corresponding terms with the Amigo tool provided on the GO website [33]. For larger data sets and sytstematic approaches some database search algorithms for proteomic data such as MaxQuant, Proteome Discoverer and X!tandem [34,35] have implemented a GO-term annotation step. As not all protein entries are fully annotaed with the corresponding GO terms, it is possible to retrieve GO-terms from the closest related protein via BLAST similarity search in the BLAST2GO tool [36].

The first step after GO-term annotation is a GO-term enrichment analysis to compare the abundance of specific GO-terms in the dataset with the natural abundance in the organism or a reference dataset, e.g. different cell lines, inhibitor treatment or growth states [37]. To extract functions that are significantly enriched in one sample over a second dataset, a p-value is calculated based which shows overrepresentation of a specific GO term, thereby it is necessary to cluster related GO-terms. This calculation can be done by most of the previously mentioned programs, but there is a plethora of other, mostly web-based software tools available ([38]http://neurolex.org/wiki/Category:Resource:Gene_Ontology_Tools). For instance, the DAVID and Babelomics software resources are often mentioned when it is necessary to analyze large gene list but currently there are more than 60 tools calculating GO term enrichment [38-40]. Most of these tools can be classified into three different types of enrichment algorithms, with singular enrichment analysis (SEA) being the most simple algorithms that test one anotation term at a time for a list of interesting genes [41]. GOStat, BinGO, or EasyGO are based on SEA algorithms. More sophisticated algorithms are gene set enrichment algorithms (GSEA) that take all genes of analysis into account, not only gene with significant change of abundance. Nevertheless, GSEA requires a quantitative measurement to rank the genes and is used in GSEA/P-GSEA and Gene Trail. Finally, modular enrichment analysis (MEA) include relationships between anotation terms which prevents loss of important biological correlations due to lacking relationships and reduces redundancy [41]. Those programs are not only limited to GO term enrichment, but they have also modules to search for protein networks (see below), convert protein identifiers, as well as link to further information and publications that substantiate the observed gene function. Especially the DAVID software resources offer a plethora of other tools for instance for gene and anotation term clustering, mapping of genes to pathways and diseases as well as advanced statistics. A second important choice for the result of GO term enrichment is the reference dataset, which is either predefined by the tool, for instance all genes of the organism, or can be selected manually (all identified proteins) [42]. Weinert et al. have applied the DAVID GO term enrichment algorithm to study conservation of acetylation sites between human and drosophila from the extracted GO-terms of acetylated proteins [43]. In their study, they showed the conservation of protein acetylation in the respiratory chain, translational processes, but also in ubiquitinating enzymes. Bates et al. could show that the Abl-kinase dependent reprogramming of B-cells is to a major part post-transcriptionally regulated, by comparing the abundance of mRNA levels with protein abundance upon imatinib inhibitor treatment [44]. Despite the usefulness of GO terms for a functional annotation and filtering of large proteomic data sets the assignement is highly dependent on the algorithm used for annotation. Recently, fourteen GO enrichment algorithms have been tested on the same dataset. The obtained results showed a rather high discrepancy for p-values of certain GO terms [42].

### Pathway analysis

A pathway describe the series of chemical reactions in the cell that lead to an observable biological effect. Proteins involved in the chemical reaction and those that have regulatory influence are combined in so-called pathway databases. Similarly to the previously described GO term enrichment analysis, protein or gene lists can also be scrutinized for pathway abundances which might be more meaningful because it moves the data interpretation away from the gene-centric view towards the identification of functional biological processes. Furthermore, functionally independent proteins can share some GO term associations, for instance for very general terms such as "binding" or "cytoplasmic". A high number of resources and databases is available to extract pathway constraints from biological data (Figure 1). Comprehensive pathway databases such as KEGG, Reactome, Ingenuity Pathway Knowledge Base or BioCarta include a high number of diverse interaction data, which could arise from intracellular reactions such as metabolism or signaling pathways, genetic interactions or drug development studies [45-47]. Apart from the comprehensive resources, highly specific databases have been developed for signal transduction processes such as PANTHER, GenMAPP or PID [48-50]. Recently, several databases were created which comprise pathways active in cancer. Such databases like Netpath[51], should help to identify cancer relevant proteins and genes from a complex dataset. In fact, public databases share a high degree of connectivity, allowing rapid distribution of novel findings. A comprehensive list of more than 300 pathway and interaction data resources can be found on the *pathguide *website http://pathguide.org[52]. Nowadays enrichment analyses are available with almost all pathway database resources, so that a list of significantly altered proteins, with regard to abundance and/or post-translational modifications, is sufficient to extract data on pathway abundance. However, similar to the GO term annotation the identification of pathways affected under certain conditions is highly dependent on the algorithm. Müller and colleagues published a comparison of the Ingenuity Pathway Analysis (IPA) software and String for the analysis of several artificial datasets [53]. The tested datasets consisted of core proteins and associated proteins of 5 different pathways, Wnt, App, and Ins signaling, mitochondrial apoptosis as well as tau phosphorylation, respectively, which were retrieved from literature mining and a set of background proteins from proteomic analysis of HEK293 cells that that were falsely annotated as significantly regulated proteins in several repeats. They reported similar results for both pathway analysis algorithms, but also that neither algorithm could reach a sufficient p-value for reliable pathway enrichment. Additional features in IPA, such as annotation of protein family and localization, might help the experienced researcher to identify falsely annotated protein hits.

### Analysis of protein-protein-interactions

The majority of proteins do not act as independent entities. They form either transient or stable complexes with other proteins that act as scaffolds or regulate the protein activity. A protein can be involved in mulitiple complexes of varying composition and to completely understand a biological system it is necessary to analyze the abundant protein complexes as well as the conditions that lead to their formation or dissociation. Information on protein interactions in complexes is deposited in interaction databases such as MINT, BioGRID, IntAct or HRPD [54-57], associated with the biological process in which they are functionally important. Not all annotated interactions in public databeases are based on experimental observations. Dependent on the database used one can find a rather high percentage of predicted interactions and interactions based on literature mining such as STRING or iRefWeb [37,58,59]. For this purpose, a variety of literature mining tools to screen PubMed abstracts has been developed of which chilibot and sciminer are most favored [60,61]. These interactions are the result of sophisticated algorithms that are trained on the existing set of protein-protein interactions. Furthermore, most large interaction databases have implemented simple algorithms that allow mapping of interaction proteins on the resource website.

Protein interactions are often displayed as large interaction networks illustrating the high degree of connectivity nand the presence of promiscous hub proteins. A widely used resource for interaction data is STRING, which is not only a database itself, but connects to several other data resources to and is therefore also capable of literature mining [59,62]. Further, STRING is also capable of drawing simple protein networks based on the provided gene list and the available interactions in its databases. Cytoscape has evolved as a powerful graphical tool to draw interaction networks of high complexity and for incorporation and comparison of datasets from different experimental procedures. Cytoscape has only limited information stored, but interconnects excessively to other databases to obtain information. Recently, EnrichNet was launched, a web-based platform integrating pathway and interaction analysis in 6 different databases (KeGG, BioCarta, Gene Ontology, Reactome, Wiki and NCI pathways) with functional associations and connecting these data with molecular function (Interpro) and protein complex information (Corum) [63]. This tool creates pathway lists and highly interactive function maps, which can also be downloaded and visualized in cytoscape. A study of the targets of cullin-ring dependent ubiquitination revealed that a large fraction of the observed proteins become modified upon activity of the SCF complex [64]. Analysis of the obtained list of SCF regulated proteins by cytoscape revealed a high degree of interconnectivity.

### Protein domain and motif analysis

When working in not yet or just recently-sequenced organisms, data bases might not contain the complete set of protein descriptions. Similarly, proteins of unknown function might also be identified from highly curated databases of well studied organisms. Those proteins often lack the previously described information on interactions and pathway affiliations so that they would not be found in such studies. To learn more about the function of those proteins and how they interact with members of certain pathways, it is helpful to analyze their amino acid sequence for specific folds of protein domains or for motifs for post-translational modifications. The simplest analysis represents a BLAST search against the database of known protein sequences to find if proteins with similar amino acid sequences have been described in other organisms [65]. Further, the amino acid sequence can be analyzed by programs such as Pfam, Interpro, SMART or also DAVID, to learn if the identified protein shares a specific protein fold with other proteins [39,66-68]. These algorithms apply hidden Markov models (HMMs) to classify proteins on basis of their amino acid sequence and predict the occurrence of a specific protein domain. Knowing about the abundance of a specific fold, could help to implement unknown proteins into biological networks. Secondly, algorithms such as MotifX or PhosphoMotif Finder analyze the sequence environment of post-translational modification sites [69,70], thereby reporting enrichment of certain amino acid motifs which can help to identify the modifying enzyme.

## Conclusions

The development of methods to systematically study all proteins in a cell and their subsequent functional annotation opens up new pathways of research. In the future it is very likely that such studies will uncover new principles of how biological systems operate hopefully leading to an improved treatment of human pathologies.

Over the last ten years the analytical harware has reached a level of sophistication of a more mature scientifc field. However, the bioinformatic interpretation and the processing of the data are still in its infancy. Besides reliable and robust algorithms, international standards for data processing and deposition as well as their interpretation have to be developed and agreed upon in order to unleash the full potential of proteomic research.

## Authors' contributions

AS, IF and AI wrote subsections of the paper and were involved in assembling the manuscript

## Competing interests

The authors declare that they have no competing interests.

## Declarations

The publication costs for this article were partly funded by a grant from the European Union (STATEGRA, 257082) and partly supported by COST-BMBS, Action BM1006 "next Generation Sequencing Data Analysis Network", SeqAhead.

This article has been published as part of BMC Systems Biology Volume 8 Supplement 2, 2014: Selected articles from the High-Throughput Omics and Data Integration Workshop. The full contents of the supplement are available online at http://www.biomedcentral.com/bmcsystbiol/supplements/8/S2.
